# Entanglement Entropy of Free Fermions with a Random Matrix as a One-Body Hamiltonian

**DOI:** 10.3390/e26070564

**Published:** 2024-06-30

**Authors:** Leonid Pastur, Victor Slavin

**Affiliations:** 1 Department of Mathematics, King’s College, London WC2R 2LS, UK; 2 B. Verkin Institute for Low Temperature Physics and Engineering, 61103 Kharkiv, Ukraine; slavin@ilt.kharkov.ua

**Keywords:** entanglement, entanglement entropy, free fermions, area law, enhanced area law, volume law, random matrices

## Abstract

We consider a quantum system of large size *N* and its subsystem of size *L*, assuming that *N* is much larger than *L*, which can also be sufficiently large, i.e., 1≪L≲N. A widely accepted mathematical version of this inequality is the asymptotic regime of successive limits: first the macroscopic limit N→∞, then an asymptotic analysis of the entanglement entropy as L→∞. In this paper, we consider another version of the above inequality: the regime of asymptotically proportional *L* and *N*, i.e., the simultaneous limits L→∞,N→∞,L/N→λ>0. Specifically, we consider a system of free fermions that is in its ground state, and such that its one-body Hamiltonian is a large random matrix, which is often used to model long-range hopping. By using random matrix theory, we show that in this case, the entanglement entropy obeys the volume law known for systems with short-range hopping but described either by a mixed state or a pure strongly excited state of the Hamiltonian. We also give streamlined proof of Page’s formula for the entanglement entropy of black hole radiation for a wide class of typical ground states, thereby proving the universality and the typicality of the formula.

## 1. Introduction

Quantum entanglement, a special form of quantum correlation, is regarded as an important ingredient of modern quantum mechanics and adjacent fields of science and technology. In its simplest form, the entanglement causes two quantum objects (spins, qubits, etc.) to share a common pure state, in which they do not have pure states of their own.

A general version of this simplest form is known as the bipartite setting, where a quantum system S consists of parties B and E, i.e.,

(1) S=B∪E.

Sometimes, these parties are two communicating agents: sometimes one of them, say, B (block), is the system of interest, while E is the environment of B, etc. There is a variety of versions and models for this general setting and related problems: see, e.g., [1,2,3,4,5,6,7,8,9,10,11,12] for reviews.

We denote by $H_{S}$, $H_{B}$, and $H_{E}$ the corresponding state spaces, so that

(2) $$H_{S}=H_{B}⊗H_{E},$$

and by ${\mathrm{tr}}_{B}$ and ${\mathrm{tr}}_{E}$ the operation of (partial) traces in $H_{B}$ and $H_{E}$. Let $\rho_{S}$ be the density matrix of S, which is often assumed to be in a pure state, i.e.,

(3) $$\rho_{S}=|\Psi_{S}\rangle \langle \Psi_{S}|.$$

Applying ${\mathrm{tr}}_{E}$ to $\rho_{S}$, we obtain the reduced density matrix

(4) $$\rho_{BS}={\mathrm{tr}}_{E}\;\rho_{S},\;{\mathrm{tr}}_{B}\;\rho_{BS}=1$$

of the B, a positive definite operator acting in $H_{B}$. This can be viewed as quantum analog of the marginal distribution of probability theory.

If B consists of several (1, 2, etc.) elementary objects, then the corresponding reduced density matrices are known in quantum statistical mechanics as the one-, two-, etc., point correlation functions. In this paper, we will deal with extended systems and their subsystems (parties), hence with reduced density matrices (correlation functions) of large size.

One of widely used numerical characteristics (quantifiers) of the quantum correlations between the parties is the entanglement entropy

(5) $$S_{BS}=-{\mathrm{tr}}_{B}\rho_{B}\log_{2}\rho_{BS},$$

i.e., the von Neumann entropy of the reduced density matrix (4).

Let Ω and Λ⊂Ω be the spatial domains occupied by S and B, and let *N* and *L* be the parameters determining the size of S and B (e.g., the corresponding side lengths if Ω is a cube in $R^{d}$ and Λ is a sub-cube, so that $\left|\Omega \right|=N^{d}$ and $\left|\Lambda \right|=L^{d}$). We will assume that

(6) 1≪L≲N,

i.e., that E (an “environment”) is much larger than B (block), which can also be sufficiently large.

The goal is to find the asymptotic form of $S_{B}$ in a certain formalization of the heuristic inequalities (6).

One of possible formalizations of (6) is as follows. The r.h.s. of (6) is implemented in its strong form L≪ N via the macroscopic limit N→∞ for S in (1), keeping *L* fixed under a condition guaranteeing the existence of a well-defined limiting entanglement entropy:

(7) $$S_{B}=\lim_{N\to \infty }S_{BS}.$$

Then, the l.h.s. 1≪L of (6) is implemented as the asymptotic regime L→∞ for $S_{B}$, i.e., shortly,

(8) firstN→∞,thenL→∞.

This asymptotic regime of the *successive* limits is natural for the systems with short-range interaction and/or hopping and has been considered in a variety of works dealing with models of quantum gravity, quantum field theory, quantum statistical mechanics, and quantum information science: see, e.g., [1,2,3,5,8,9,12,13,14,15,16] for reviews. It has been found on various levels of rigor that in the translation-invariant case, the leading term of the large-*L* asymptotic of the macroscopic limit (7) of the entanglement entropy (5) can be as follows:

(i) the area law

(9) $$S_{B}=C_{d}^{\prime }\;L^{d-1}\left(1+o\left(1\right)\right),\;L\to \infty ,$$

if S is in its non-critical ground state (no quantum phase transition), or/and if there is a spectral gap between the ground state and the rest of the spectrum;

(ii) the enhanced (violation of) area law

(10) $$S_{B}=C_{d}^{\prime \prime }\;L^{d-1}\log L\left(1+o\left(1\right)\right),\;L\to \infty ,$$

if S is in its critical ground state (a quantum phase transition is present);

(iii) the volume law

(11) $$S_{B}=C_{d}^{\prime \prime \prime }\;L^{d}\left(1+o\left(1\right)\right),\;L\to \infty ,$$

if S is either in a mixed state, say, the Gibbs state of non-zero temperature, or in a pure but sufficiently highly excited state, with the latter case being closely related to the fundamental Entanglement Thermalization Hypothesis [1,7]. Note that the coefficients $C_{d}^{\prime },C_{d}^{\prime \prime }$, and $C_{d}^{\prime \prime \prime }$ do not depend on *L*.

Certain disordered quantum systems have also been considered, mainly various spin chains, and both the one-dimensional area law and the enhanced area law have been found and analyzed: see, e.g., [8,10,13,17,18] and the references therein.

Arguments establishing the above results turn out to be rather involved and are not always sufficiently transparent and undoubted, especially in the multi-dimensional case. This is why the rather simple but non-trivial model of free fermions living on the lattice $Z^{d}$ has attracted considerable attention: see, e.g., [15,19,20,21,22,23,24] and the references therein.

This model is described using the quadratic many-body Hamiltonian

(12) $$\sum_{m,n\in \Omega }H_{mn}c_{m}^{+}c_{n},$$

where ${\left\{c_{m},c_{m}^{+}\right\}}_{m\in \Omega },\;c_{m}^{+}c_{n}+c_{n}c_{m}^{+}=\delta_{mn}$ are the annihilation and creation operators of free spinless fermions and

(13) $$H_{S}={\left\{H_{mn}\right\}}_{m,n\in \Omega }$$

is their one-body Hamiltonian. Note that $H_{S}$ acts in the $\left|\Omega \right|=N^{d}$-dimensional complex Euclidean space $C^{\left|\Omega \right|}$, while (12) acts in the much “bigger” space $H_{S}$ of dimension $2^{\left|\Omega \right|}$: see (2). The entries $\left\{H_{mn}\right\}$ of $H_{S}$ are sometimes called hopping parameters.

It should be noted that the bipartite setting based on the form (2) of the state space, which is widely used in quantum information science (dealing with qubits) and quantum statistical physics (dealing with spins), is not directly applicable to indistinguishable particles, fermions in particular. Therefore, in this case, one proceeds not from states (see (2)), but from the algebra of observables of the entire system and that (local) of its subsystems generated through the creation and annihilation operators in the coordinate representation of the second quantization: see, e.g., [11,12,16] for reviews.

An important component that strongly facilitates the analysis of the entanglement entropy of free fermions is a convenient formula for the $S_{BS}$ of (5), expressing it via the so-called Fermi projection of the one-body Hamiltonian (13): see, e.g., [15,17,25] and Formulas (18) and (19) below. The formula for this is as follows:

Given that −∞≤a<b≤∞, denote

(14) $$\chi_{\left(a,b\right)}\left(\varepsilon \right)=\left\{\begin{array}{cc} 1, & \varepsilon \in (a,b),\\ 0 & \varepsilon \in R\setminus (a,b), \end{array}\right.$$

the indicator of (a,b). Then,

(15) $$P_{S}=\chi_{\left(-\infty ,\varepsilon_{F}\right)}\left(H_{S}\right)$$

is the Fermi projection of $H_{S}$ and $\varepsilon_{F}$ is the Fermi energy (a free parameter). In other words, $P_{S}$ is the orthogonal projection on the subspace of the one-body state space $C^{\left|\Omega \right|}$ spanned by the eigenvectors ${\left\{\psi^{\alpha }\right\}}_{\alpha =1}^{N}$ of $H_{S}$, with eigenvalues ${\left\{\varepsilon_{\alpha }\right\}}_{\alpha =1}^{N}$ belonging to $\left(-\infty ,\varepsilon_{F}\right]$; hence,

(16) $$P_{S}={\left\{P_{m_{1}m_{2}}\right\}}_{m_{1},m_{2}\in \Omega },\;P_{m_{1}m_{2}}=\sum_{\varepsilon_{\alpha }\in \left[\varepsilon_{0},\varepsilon_{F}\right)}\psi_{m_{1}}^{\alpha }\;\bar{\psi_{m_{2}}^{\alpha }}.$$

Let

(17) $$P_{BS}={\left\{P_{l_{1}l_{2}}\right\}}_{l_{1},l_{2}\in \Lambda }$$

be the restriction of $P_{S}$ to Λ⊂Ω. Then, we have the following formula [15,25]:

(18) $$S_{BS}={\mathrm{Tr}}_{L}h\left(P_{BS}\right),$$

where

(19) $$h\left(x\right)=-x\log_{2}x-\left(1-x\right)\log_{2}\left(1-x\right),\;x\in \left[0,1\right]$$

(the binary Shannon entropy), and ${\mathrm{Tr}}_{\Lambda }$ is the “partial” trace in $C^{\left|\Lambda \right|}\subset C^{\left|\Omega \right|}$ (do not confuse this with ${\mathrm{tr}}_{B}$ in (4) and (5), or the trace operation in the $2^{\left|\Lambda \right|}$-dimensional state space $H_{B}$ of the block in (2)).

The Formula (18) reduces the analysis of the entanglement entropy of free fermions to the spectral analysis of the one-body Hamiltonian $H_{S}$ of (12) and (13).

One more interesting aspect of this formula is that it provides a link with the studies of asymptotic trace formulas for various classes of matrix and integral operators, in particular the so-called Szegö’s theorem and its generalizations: see, e.g., [22,26,27,28] and the references therein.

It is usually assumed that there exists a well-defined infinite-volume Hamiltonian *H* (cf. (7)),

(20) $$H:=\lim_{N\to \infty }H_{S},$$

in a certain sense. In fact, this assumption is a weak form of the requirement for the one-body Hamiltonian to exhibit short-range hopping and is quite natural in the regime (8). It follows then from the variety of works that in the translation-invariant case with a short-range hopping *H* (e.g., the discrete Laplacian), the leading term of the asymptotic formula for the entanglement entropy in the regime (8) has again one of the three forms described in (9)–(11).

Namely, it is the area law (9) if the Fermi energy $\varepsilon_{F}$ is in a gap of the spectrum of *H* in (20), and it is the enhanced area law (10) if $\varepsilon_{F}$ is in the spectrum of *H* and the system is in its ground state, i.e., at zero temperature. If, however, T>0, and hence the indicator $\chi_{\left(-\infty ,\varepsilon_{F}\right)}$ (see (14)) is replaced in (15) by the Fermi distribution

(21) $${\left(1+e^{(\varepsilon -\varepsilon_{F})/T}\right)}^{-1},\;T>0,$$

or, more generally, just by a continuous function, then we have the volume law (11): see, e.g., [3,13,14,29] for reviews.

For disordered free fermions, where the one-body Hamiltonian is a discrete Schrodinger operator with random potential (Anderson model), i.e., for the disordered short-range hopping case, the validity of all three asymptotic Formulas (9)–(11) for the entanglement entropy has been rigorously established in [20,24,26,30]. However, in this case, the area law is valid not only if the Fermi energy $\varepsilon_{F}$ is in the gap of the spectrum of *H* in (20), but also if $\varepsilon_{F}$ is in the localized part of the spectrum. As for the validity of the enhanced area law, this is the case if the Fermi energy coincides with a so-called transparency energy of *H*: see [30] for this result and Section 10.3 of [31] for the definition and properties of transparency energies.

In addition, certain new properties of the entanglement entropy were found in the disordered case: the vanishing of the fluctuations of the entanglement entropy (self-averaging) for d≥2 as L→∞ [20]; non-trivial fluctuations for d=1 [24]; the Central Limit Theorem for entanglement entropy at non-zero temperatures for d=1 (see (21)); and, as a result, the $L^{1/2}$ (instead of $L^{0}$) scaling of the sub-leading term for the volume law for d=1 [24].

As already mentioned, the above asymptotic results for both spin systems and free fermions were obtained in the successive-limits regime (8). On the other hand, one can consider the implementations of heuristic inequalities (6), where *N* and *L* tend to infinity simultaneously:

(22) $$N\to \infty ,\;L\to \infty ,\;L/N^{\alpha }\to \lambda_{\alpha }>0,\;\alpha \in \left(0,1\right],\;\lambda_{1}\in \left(0,1\right],$$

e.g.,

(23) $$L=\left[\lambda_{\alpha }N^{\alpha }\right]=\lambda_{\alpha }N^{\alpha }+O\left(1\right),\;N\to \infty .$$

Both asymptotic regimes (8) and (22)–(23) are of interest in view of the general bipartite setting (1)–(6) of quantum information theory. We outlined above a collection of results on the asymptotics of the entanglement entropy of extended quantum systems with local interaction and/or hopping obtained in the frameworks of the regime (8) of successive limits. The regime (22)–(23) of simultaneous limits (or the double scaling limit) is also of interest in the local case, for example in order to probe the “amount” of universality of the enhanced area law (10), i.e., the maximal α in (22)–(23) up to which the law holds. This is of interest both in itself and because it seems more adequate to a wide variety of numerical studies of extended systems, where it is often hard, if not impossible, to appropriately implement the successive limits (8). The same regime is quite common in non-equilibrium and quantum chaos studies: see, e.g., the review paper in [29] and the references therein. It is also quite instrumental in the branch of random matrix theory dealing with sample covariance matrices and in related branches of multivariate statistics (big data, mathematical finance, etc.): see [32], Chapters 7 and 19, and the references therein.

In this paper, we consider the regime (22) and (23) for the systems of free fermions where $H_{S}$ is the N×N hermitian random matrix with a unitary invariant probability law, e.g., the well-known Gaussian Unitary Ensemble (GUE): see [32] for results and references. It is widely believed and has been confirmed by various recent results (see, e.g., [32,33]) that large random matrices may model multi-component and multi-connected media, playing the role of the mean field-type approximation for the Schrodinger operator with random potential, a basic model in the theory of disordered systems and related branches of spectral theory and solid-state theory. We will show that in the case of this (long-range) one-body Hamiltonian, the entanglement entropy obeys the volume law (11) with d=1, as seen in Result 3.

The corresponding results are presented in Section 2.1 and Section 2.2 and are proved in Appendix A, Appendix B, Appendix C and Appendix D.

In Section 2.3 and Appendix E, we deal with a related problem, although it does not involve free fermions. This problem was initially considered in the context of quantum gravity, where E and B in (1) play the role of a black hole in the pure initial state of the evaporation process and outgoing Hawking radiation, respectively [9,34,35]. The idea was that the generic evaporative dynamics of a black hole may be captured through the random sampling of subsystems of a quantum system that is in a pure random initial state.

This was one of the first applications of random matrices to cosmology that prompted extensive activities covering several fields: see, e.g., [2,6,9,29,36,37].

It is also worth mentioning that there is a link between the results of [9,34,35] and the asymptotic Formulas (9)–(11), especially with the volume law (see Section 2.3).

## 2. Results

We present here our results and their discussions. The corresponding technical proofs are given in Appendix A, Appendix B, Appendix C, Appendix D and Appendix E.

### 2.1. Generalities

To study possible asymptotic formulas for the entanglement entropy (18) of free fermions at zero temperature, we will use general bounds given by the following:

**Result 1.**  *Given the general setting (12)–(19) for the model of free lattice fermions, we have the following bounds for the entanglement entropy $S_{SB}$ (5):*

(24) $$L_{SB}\leq S_{SB}\leq U_{SB},$$

*where*

(25) $$\begin{array}{cc} \; & L_{SB}=4{\mathrm{Tr}}_{\Lambda }P_{SB}\left(1_{\Lambda }-P_{SB}\right)=\sum_{l\in \Lambda ,k\in \Omega \setminus \Lambda }{\left|P_{lk}\right|}^{2},\\ \; & \;U_{SB}=|\Lambda |\;h_{0}(L_{SB}/4|\Lambda |), \end{array}$$

*and*

(26) $$h_{0}:\left[0,1/4\right]\to \left[0,1\right],\;h\left(x\right)=h_{0}\left(x\left(1-x\right)\right),\;x\in \left[0,1\right],$$

*with H given by (19).*

*If the one-body Hamiltonian $H_{S}$ is random, then (24) and (25) are valid for every realization, while we have the following for the expectation $E\{S_{SB}\}$ of $S_{SB}$:*

(27) $${\bar{L}}_{SB}\leq E\left\{S_{SB}\right\}\leq {\bar{U}}_{SB},$$

*where*

(28) $${\bar{L}}_{SB}=E\left\{L_{SB}\right\},\;{\bar{U}}_{SB}=\left|\Lambda \right|h_{0}({\bar{L}}_{SB}/4|\Lambda |).$$

**Figure 1:**
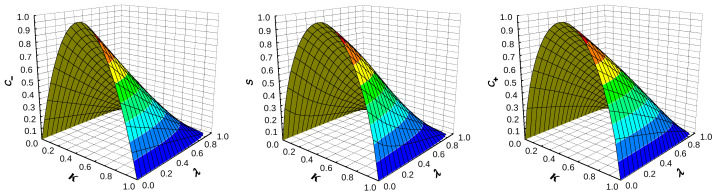
The coefficients $C_{-}$ of (48) (**left panel**), $s_{\kappa \lambda }$ of (53)–(54) (**central panel**), and $C_{+}$ of (48) (**right panel**) as functions of parameters κ of (40) and λ of (23).

**Figure 2:**
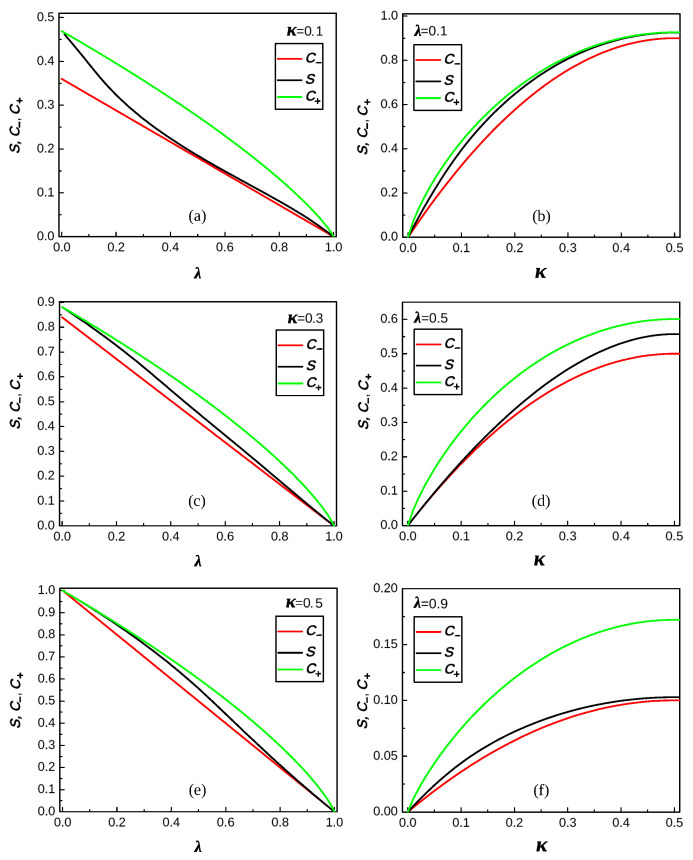
**Left** (**a**,**c**,**e**): The coefficients $C_{-}$ (red) and $C_{+}$ (green) of (48), and $s_{\kappa \lambda }$ (black) of (53) as functions of λ for different values of κ. **Right** (**b**,**d**,**f**): The same coefficients as functions of κ for different values of λ.

**Figure 3:**
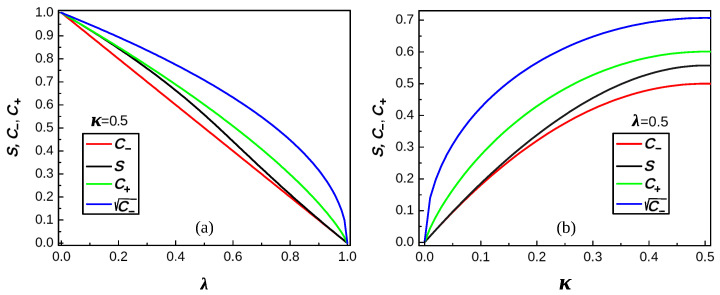
The same as in Figure 2, with the coefficient $\sqrt{C_{-}}$ (blue) of (57).

These bounds are proven in Appendix A. They allow us to obtain, by using an elementary argument and technique, rather tight bounds for the entanglement entropy: see (46), (48), (50), Result 2, and the materials below, including Table 1 and Figure 1, Figure 2e and Figure 3a.

The same bounds are used in [38] to study the enhanced area law (10) for translation-invariant free fermions in the regime (22) and (23).

Note also that the lower bound $L_{SB}$ in (24) and (25) is proportional to the variance of the number of fermions in Λ: see, e.g., [18] and the references therein. This quantity can also be expressed via the density–density correlator ${\left(\delta \left(E^{\prime }-H_{S}\right)\right)}_{mn}{\left(\delta \left(E^{\prime \prime }-H_{S}\right)\right)}_{nm}$, important in the solid-state theory [31,39].

For other versions of two-sided bounds for the entanglement entropy, see [13,40,41,42] and the references therein.

We will also use the spectral version of the basic Formulas (18) and (19). Let

(29) $$N_{P_{BS}}=\sum_{\alpha =1}^{\left|\Lambda \right|}\delta_{p_{\alpha }}$$

be the counting measure of eigenvalues ${\left\{p_{\alpha }\right\}}_{\alpha =1}^{\left|\Lambda \right|}$ of $P_{BS}$ of (17). Then, we can write (18) as

(30) $$S_{BS}=\int_{0}^{1}h\left(p\right)N_{P_{BS}}\left(dp\right).$$

This reduces the asymptotic study of $S_{BS}$ to that of $N_{P_{BS}}$. The latter is often not simple to find (see, however, [43], as well as (A23) and (A24), and (A7) and (54) below), but formula (30) proves to be also useful for interpreting various results on the entanglement entropy of free fermions.

### 2.2. Entanglement Entropy of Free Fermions with a Random-Matrix Hamiltonian

We will assume here that the whole system S and its block B occupy the integer valued intervals

(31) Ω=(1,2,…,N),Λ=(1,2,…,L).

It is convenient at this point to change the notation and write subindices *N* and *L* instead of S and B:

(32) S→N,B→L.

We will assume then that the one-body Hamiltonian (13) is

(33) $$H_{N}=M_{N},$$

where $M_{N}$ is the N×N hermitian random matrix, whose probability law is invariant with respect to all unitary transformations $M_{N}\to U_{N}M_{N}U_{N}^{*},\;U_{N}\in U\left(N\right)$. An interesting and widely studied subclass of this class of random matrices consists of the so-called matrix models (also known as invariant ensembles), where the matrix probability law is

(34) $$\begin{array}{cc} \; & Z_{N}^{-1}e^{-N\mathrm{Tr}V(M_{N})}dM_{N},\\ V(x) & \geq \left(1+\varepsilon \right)\log\left(1+x^{2}\right),\;\varepsilon >0,\;x\in R,\\ \; & \;dM_{N}=\prod_{1\leq n\leq N}dM_{nn}\prod_{1\leq n_{1}<n_{2}\leq N}dℜM_{n_{1}n_{2}}dJM_{n_{1}n_{2}}, \end{array}$$

as seen in, e.g., [32,44].

The most known example of matrix models is the Gaussian Unitary Ensemble (GUE), where $V\left(x\right)=2x^{2}/\varepsilon_{0}$. In this case, the entries of $M_{N}$ are complex Gaussian random variables:

(35) $$\begin{array}{cc} M_{N} & =\varepsilon_{0}{\left(4N\right)}^{-1/2}{\left\{X_{m_{1}m_{2}}\right\}}_{m_{1},m_{2}=1}^{N},\\ E\{X_{m_{1}m_{2}}\} & =E\left\{X_{m_{1}m_{2}}^{2}\right\}=0,\;E\{|X_{m_{1}m_{2}}{|}^{2}\}=1. \end{array}$$

Thus, all entries of $M_{N}$ in (34) and (35) have the same order of magnitude ($N^{-1/2}$ for the GUE); hence, the limiting operator *H* of (20) does not exist in this case. This should be contrasted with the short-range hopping case where the limiting operator is well defined and is a discrete Laplacian in the simplest case of lattice translation-invariant fermions, and a Schrodinger operator with random potential (Anderson model) for disordered free fermions, both acting in $l^{2}\left(Z^{d}\right)$: see, e.g., [20] and the references therein.

An analogous situation is in the mean field models of statistical mechanics. On the other hand, a number of important characteristics have well-defined macroscopic limits (e.g., the free energy in statistical mechanics, and the limiting Normalized Counting Measure in random matrix theory). This allow us to view (33) as a disordered version of the mean field model for free fermions, and to expect that the entanglement entropy will exhibit a well-defined asymptotic behavior in this case.

Note that $M_{N}$ of (35), more precisely its real symmetric analog (GOE), is used as the interaction matrix in a highly non-trivial mean field model of spin glasses known as the Scherrington–Kirkpatrick model [45], where the the role of Fermi operators in (12) play classical or quantum spins. This model is a disordered version of the well-known Kac model, where the interaction matrix is $\varepsilon_{0}N^{-1}{\left\{1\right\}}_{m_{1},m_{2}=1}^{N},$ $\varepsilon_{0}>0$ and the corresponding spin model reproduces the well-known Curie–Weiss description of the ferromagnetic phase transition in the large-*N* limit.

By the way, by using the “Kac” interaction matrix $\varepsilon_{0}N^{-1}{\left\{1\right\}}_{m_{1},m_{2}=1}^{N},\;\varepsilon_{0}>0$ as the one-body Hamiltonian in (12), it is easy to find that the corresponding entanglement entropy is independent of *L*. Indeed, we can write

(36) $$H_{N}=\varepsilon_{0}P_{d_{\Omega }},$$

where $P_{d_{\Omega }}$ is the orthogonal projection on the “diagonal” vector $d_{\Omega }={\left|\Omega \right|}^{-1/2}\left\{1,\ldots ,1\right\}\in C^{\left|\Omega \right|}$.

Then, the corresponding Fermi projection is as follows (see (15) and (16) and (32)):

$$P_{N}=\chi_{\left(-\infty ,\varepsilon_{F}\right)}\left(0\right)\left(1_{\Omega }-P_{d_{\Omega }}\right)+\chi_{\left(-\infty ,\varepsilon_{F}\right)}(\varepsilon_{0})P_{d_{\Omega }},$$

where $\chi_{\left(-\infty ,\varepsilon_{F}\right)}$ is the indicator of $(-\infty ,\varepsilon_{F})\subset R$ (see (14)), and the restriction (17) of $P_{\Omega }$ to Λ=[1,…,L] is

$$P_{LN}=\chi_{\left(-\infty ,\varepsilon_{F}\right)}\left(0\right)\left(1_{\Lambda }-L/NP_{d_{\Lambda }}\right)+\chi_{\left(-\infty ,\;\varepsilon_{F}\right)}\left(\varepsilon_{0}\right)L/NP_{d_{\Omega }}.$$

It follows then from (18) that

(37) $$S_{LN}=h\left(L/N\right)=h\left(1-L/N\right).$$

Hence, the entanglement entropy is zero in the regime (8) of successive limits (moreover, $S_{\Lambda }$ of (7) is already zero), and in the regime (22) of simultaneous limits if α<1. On the other hand,

(38) $$\lim_{N\to \infty ,L\to \infty ,L/N\to \lambda_{1}}S_{LN}=h\left(1-\lambda_{1}\right)=h\left(\lambda_{1}\right)$$

in the regime (22) with α=1, i.e., for an asymptotically proportional |Λ|=L and |Ω|=N.

Formula (38) corresponds formally to the one-dimensional area law (9), although the notion of surface is not well defined in the mean field setting.

We will show now that for random matrices (34), a disordered version of the Kac model, the situation is in some sense “opposite”, since in this case the entanglement entropy obeys the analog of the volume law (11).

To this end, we note first that because of the unitary invariance of (34), the eigenvalues and the eigenvectors of $M_{N}$ are statistically independent, and the eigenvectors form a random unitary matrix $U_{N}={\left\{U_{jk}\right\}}_{j,k=1}^{N}$ that is uniformly (Haar) distributed over the group U(N) [32]. Hence, the Fermi projection (15) and (16) in this case is as follows (see (32)):

(39) $${\left(P_{N}\right)}_{m_{1}m_{2}}=\sum_{k=1}^{K}U_{m_{1}k}\bar{U_{m_{2}k}},\;m_{1},m_{2}=1,\ldots ,N,$$

where

(40) $$K=\left[N\kappa_{F}\right],\;\kappa_{F}=\nu_{M}\left(\varepsilon_{F}\right)\in \left(0,1\right)$$

$\kappa_{F}$ is the analog of the Fermi momentum fixing the ground state (the Fermi sea) of free fermions, and $\nu_{M}$ is the limiting Normalized Counting Measure of $M_{N}$ (cf. (29))

(41) $$\nu_{M}=\lim_{N\to \infty }N^{-1}N_{M_{N}}:$$

see [32,44] for the proof of (41) and various examples, the most known being the Wigner semicircle law

$$\nu_{M}^{\prime }\left(\varepsilon \right)=2{\left(\pi \varepsilon_{0}^{2}\right)}^{-1}{\left(\varepsilon_{0}^{2}-\varepsilon^{2}\right)}^{1/2}\chi_{\left[-\varepsilon_{0},\varepsilon_{0}\right]}\left(\varepsilon \right)$$

for the GUE (35) with χ defined in (14).

Furthermore, the analog of the restriction $P_{LN}$ (17) of $P_{N}$ (39) is, in this case,

(42) $${\left(P_{LN}\right)}_{l_{1}l_{2}}=\sum_{k=1}^{K}U_{l_{1}k}\bar{U_{l_{2}k}},\;l_{1},l_{2}=1,\ldots ,L.$$

We will again begin with the asymptotic bounds (24), this time for the expectation $E\{S_{LN}\}$ of the entanglement entropy (18) corresponding to (33).

To simplify the further notation, we will write below κ instead of $\kappa_{F}$ (see (40)), and λ instead of $\lambda_{1}$ (see (22) and (23), as well as (38)):

(43) $$\lambda_{1}\to \lambda ,\;\kappa_{F}\to \kappa .$$

**Result 2.**  *Let the one-body Hamiltonian $H_{N}$ of the system of free fermions be the random matrix (33). Assume that (see (40))*

(44) $$\begin{array}{cc} \; & \;N\to \infty ,\;K\to \infty ,\;L\to \infty ,\;K/N\to \kappa \in (0,1). \end{array}$$

*Then, the expectation of the entanglement entropy (see (18) and (19)) of the block (31) admits the following asymptotic bounds:*

(45) $$\begin{array}{cc} \; & \;{\hat{L}}_{LN}\leq E\left\{S_{LN}\right\}\leq {\hat{U}}_{LN},\\ \; & {\hat{L}}_{LN}=4\kappa \left(1-\kappa \right)L\left(1-L/N\right)+o\left(L\right),\\ \; & \;{\hat{U}}_{LN}=Lh_{0}\left(\kappa \left(1-\kappa \right)L\left(1-L/N\right)\right)+o\left(L\right). \end{array}$$

The proof of this result is given in Appendix B.

The final asymptotic bounds for $E\{S_{LN}\}$ are determined by the order of magnitude of *L* with respect to *N* as N→∞: see (22).

(i) N→∞,L→∞,L/N→0, i.e., 0<α<1 in (22) and (23):

(46) $$\begin{array}{cc} \; & C_{-}L+o\left(L\right)\leq E\left\{S_{LN}\right\}\leq C_{+}L+o\left(L\right),\\ \; & C_{-}=4\kappa \left(1-\kappa \right),\;C_{+}=h_{0}\left(\kappa \left(1-\kappa \right)\right). \end{array}$$

Moreover, since $C_{-}=C_{+}=1$ for κ=1/2 (see (19) and (26)), we have in this case an exact asymptotic formula:

(47) $$E\left\{S_{LN}\right\}=L+o\left(L\right),\;L\to \infty .$$

Note that these bounds are valid even for a finite *L*, but with the replacements of o(L) by o(N).

Bearing in mind that *L* plays in this case the role of the size (volume) of the block, this result can be viewed as an indication of the validity of the volume law (11) for the mean entanglement entropy, both in the regime (8) of simultaneous limits and in the regime (22) and (23) of successive limits for α<1. Note that in the translation-invariant short-range case, we have in this situation the enhanced area law (10): see [22] and the references therein.

(ii) N→∞,L/N→λ∈(0,1), i.e., α=1 in (22) and (23) (see (43)):

(48) $$\begin{array}{cc} \; & C_{-}L+o\left(L\right)\leq E\left\{S_{LN}\right\}\leq C_{+}L+o\left(L\right),\;L\to \infty \\ \; & C_{-}=4\kappa \left(1-\kappa \right)\left(1-\lambda \right),\;C_{+}=h_{0}\left(C_{-}/4\right). \end{array}$$

The bounds rule out the area law (9) and the enhanced area law (10), and are compatible only with the volume law but with coefficients that are different from those of the previous case (46). In particular, since 0<λ≤1 in general, an exact asymptotic formula (see (47)) cannot be obtained from (48).

For similar results pertinent to related matrix models and their applications, see [29].

We conclude that for the random-matrix (long-range hopping) one-body Hamiltonian (33) of free fermions, a possible asymptotic law for the expectation of the entanglement entropy in both asymptotic regimes (8) and (22)–(23) is an analog of the volume law (11).

To see the indications for other possible scalings of $E\{S_{LN}\}$, let us consider the case where

(49) $$L=N\left(1-\delta_{N}\right),\;\delta_{N}=o\left(1\right),\;N\to \infty ,$$

corresponding to the blocks with size *L* close to the size *N* of the entire system. Then, (45) implies as follows for, say, $\delta_{N}=\log N/N$:

(50) $$4\kappa \left(1-\kappa \right)\log L\left(1+o\left(1\right)\right)\leq E\left\{S_{LN}\right\}\leq \kappa \left(1-\kappa \right)\log^{2}L\left(1+o\left(1\right)\right),\;N\to \infty ,$$

and we obtain the bounds that are compatible only with the one-dimensional enhanced area law scaling (10).

For similar bounds in the translation-invariant short-range hopping case, see [13,38,42].

We will now use certain random matrix theory results to show that in the above case (ii) of the asymptotic regime (22) and (23) of simultaneous limits, the analog of the volume law is valid for all typical realizations of the entanglement entropy itself, as well as for its expectation.

**Result 3.**  *Under the conditions of the previous Result 2, i.e., for*

(51) K→∞,L→∞,N→∞,K/N→κ∈(0,1),L/N→λ∈(0,1),

*the entanglement entropy (18) and (19) of the block *Λ* of (31) admits the volume law asymptotic formula, valid with probability 1:*

(52) $$S_{LN}=Ls_{\kappa \lambda }+o\left(L\right),\;N\to \infty .$$

*Here, the coefficient (the “specific” entropy) $s_{\kappa \lambda }$ is non-random and equals*

(53) $$s_{\kappa \lambda }=\int_{p_{-}}^{p_{+}}h\left(p\right)\nu_{ac}^{\prime }\left(p\right)dp,$$

*where*

(54) $$\begin{array}{cc} \; & \nu_{ac}^{\prime }\left(p\right)=\frac{\sqrt{\left(p_{+}-p\right)\left(p-p_{-}\right)}}{2\pi \lambda p(1-p)}\chi_{\left[p_{+},p_{-}\right]}\left(p\right),\;p_{\pm }={\left(\sqrt{\kappa (1-\lambda )}\pm \sqrt{\lambda (1-\kappa )}\right)}^{2}, \end{array}$$

*with the χ defined in (14), and*

(55) $$\begin{array}{cc} \; & \;s_{\kappa \lambda }=\frac{1}{\ln(2)}\{s\frac{|\kappa -\lambda |-1+|\kappa -\bar{\lambda }|}{2\lambda }-\kappa \ln\left(2\kappa \lambda \right)\\ \; & +\bar{\kappa }\frac{\bar{\lambda }}{\lambda }\ln\left(\frac{\bar{\kappa }+\bar{\lambda }-\left|\kappa -\lambda \right|}{2\bar{\kappa }\bar{\lambda }}\right)+\kappa \ln\left(\kappa +\lambda -|\kappa -\lambda |\right)+\\ \; & +\kappa \frac{\bar{\lambda }}{\lambda }\ln\left(\frac{\kappa +\bar{\lambda }-\left|\kappa -\bar{\lambda }\right|}{2\kappa \bar{\lambda }}\right)+\bar{\kappa }\ln\left(\frac{\bar{\kappa }+\lambda -\left|\lambda -\bar{\kappa }\right|}{2\lambda \bar{\kappa }}\right)\} \end{array}$$

*with $\bar{\kappa }=1-\kappa ,\;\bar{\lambda }=1-\lambda .$*

The proof of this result is given in Appendix C.

**Remark 1.** *(i) The coefficient $s_{\kappa \lambda }$ in (52)–(55) has different analytic expressions in four sectors of the square ${\left[0,1\right]}^{2}$. They are determined by the conditions of the vanishing of atoms of the limiting Normalized Counting Measure $\nu_{ac}$ of (A6) and (A7): I: $m_{0}=m_{1}=0\;\left(\lambda <\kappa ,\;\lambda <\bar{\kappa }\right)$; II: $m_{0}=0,\;m_{1}>0\;\left(\lambda <\kappa ,\;\lambda >\bar{\kappa }\right)$; III: $m_{0}>0,\;m_{1}=0\;\left(\lambda >\kappa ,\;\lambda <\bar{\kappa }\right)$; and IV: $m_{0},\;m_{1}>0\;\left(\lambda >\kappa ,\;\lambda >\bar{\kappa }\right)$ (cf. (68)). These expressions are given in Table 1.*

**Figure 4:**
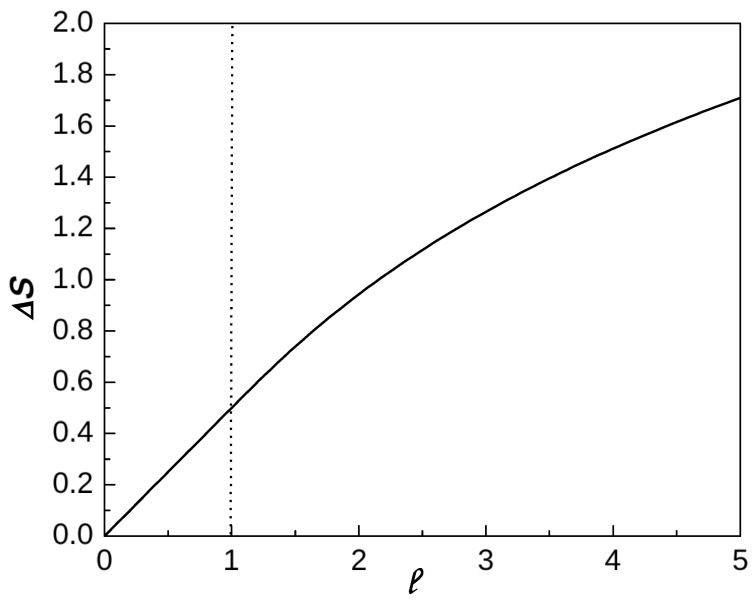
The “deficit” $\Delta =-(S_{\mathrm{LN}}-\log L)$ of (68) (and (75) and (A32)) as a function of *ℓ*.

**Figure 5:**
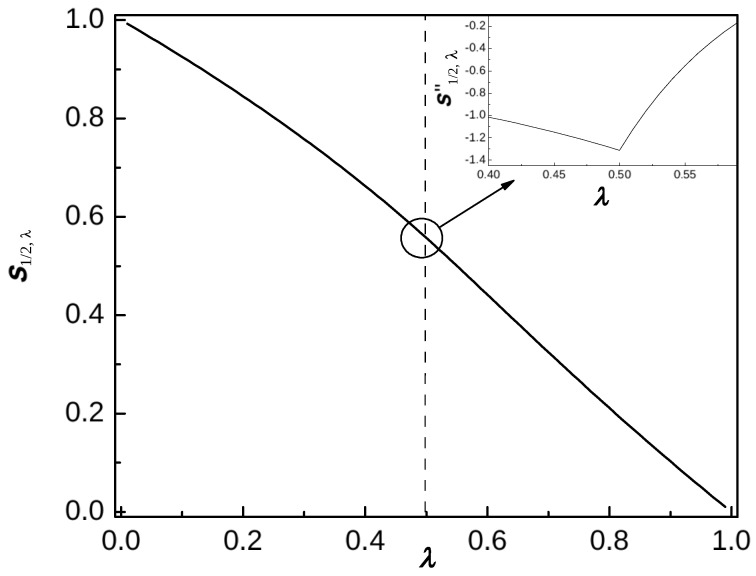
The coefficient $s_{\kappa \lambda }{|}_{\kappa =1/2}$ of (52)–(55) coinciding with the leading term of the average entanglement entropy of the model studied in [19,23]: see also Figure 2e and Figure 3a and the review in [29].

*The corresponding calculations and the result are similar to but more involved than those in Appendix E, dealing with a one-parametric analog of the above. In particular, the plot in Figure 4 of the piece-wise analytic function, given by the second term in the r.h.s. in (68) (see also (A32)), is the one-parametric analog of Figure 1 describing the surface $s_{\kappa \lambda },\;\left(\kappa ,\lambda \right)\in {\left[0,1\right]}^{2}$. For more analogs, see (56), as well as Figure 2e, Figure 3a and Figure 5.*

*(ii). A particular case κ=1/2,0<λ<1 of $s_{\kappa \lambda }$ is*

(56) $$s_{\kappa \lambda }=\left\{\begin{array}{cc} 1-\frac{1+(1-\lambda )/\lambda \ln(1-\lambda )}{\ln(2)}, & 0\leq \lambda \leq 1/2,\\ \frac{1-\lambda }{\lambda }-\frac{\left(1-\lambda )/\lambda +\ln(\lambda \right)}{\ln(2)}, & 1/2\leq \lambda \leq 1. \end{array}\right.$$

*The expressions for λ∈[0,1/2] and λ∈[1/2,1], as well as their first and second derivatives, coincide at λ=1/2; however, the third derivatives are different: see Figure 5 (cf. (75)). The part of the expression for 0<λ<1/2 coincides with the expression conjectured in formula (2) of [19] and proved in [23] as the leading term in formula (7), in the framework of a somewhat different model and with a different method.*

*To see the coincidence, one has to take into account that our definition (5) of the entanglement entropy uses $\log_{2}$ (responsible for the factor $\ln\left(2\right):=\log_{e}2$), and we assume that the Density of States (A6) (see also (A18)) of the Fermi projection contains the normalizing factor ${\left|\Lambda \right|}^{-1}=L^{-1}$.*

Because the above formulas for $s_{\kappa \lambda }$ are rather complex, we give below certain graphic and numeric results concerning $s_{\kappa \lambda }$ and the coefficients $C_{\pm }$ in bounds (48). Figure 1, Figure 2 and Figure 3 present various graphical manifestations of the proximity of $C_{-}$ and $C_{+}$ to $s_{\kappa \lambda }$ for the various pairs $\left(\kappa ,\lambda \right)\in {\left[0,1\right]}^{2}$ of the parameters κ and λ of the Hamiltonian (see (40) and (54)). Figure 1 gives the shape of three “surfaces” describing $C_{-}$, $C_{+}$, and $s_{\kappa \lambda }$, which are quite close to each other. Note that the surface of the central panel is the two-parameter analog of the piece-wise analytic curve of Figure 5, describing (68). Figure 2 gives the values of $C_{-}$, $C_{+}$, and $s_{\kappa \lambda }$ as functions of one of the parameters for certain fixed values of the other, and Figure 3 gives the same values, supplemented by those of the coefficient $C_{-}^{1/2}$ of the upper bound

(57) $$U_{\Lambda ,\Omega }^{*}={\left(LL_{\Lambda ,\Omega }\right)}^{1/2}=C_{-}^{1/2}L+o\left(L\right):$$

see [13,40,41] and the references therein concerning the bound.

Table 2 shows numerical data on the closeness of the curves of Figure 2 measured by the maximum distances between the corresponding pairs of curves.

We conclude that in the asymptotic regime (44), known in random matrix theory as the global regime, we have with probability 1 (for all typical realizations) an analog of the volume law that is quite well approximated by the bounds given in (24).

To calculate the possibility of the entanglement entropy in the random-matrix case being other than the volume law asymptotic form, let us assume that λ=1−δ with a sufficiently small (but *N*-independent) δ>0, corresponding to the blocks of size close to that of the whole system (cf. (49)). It follows then from (54) that

$$p_{\pm }{|}_{\lambda =1-\delta }=\left(1-\kappa \right)\pm 2\;\delta^{1/2}\sqrt{\kappa (1-\kappa )}+O\left(\delta \right);$$

hence, the width of the support of $\nu_{ac}^{\prime }$ is $O(\delta^{1/2})$ and we obtain in view of (52), (53) and (54):

(58) $$s_{\kappa ,1-\delta }=h\left(\kappa \right)\;\delta +o\left(\delta \right),\;\delta =o\left(1\right).$$

The last formulas can be interpreted as an indication of the possibility of obtaining the scaling L=o(N), i.e., a “subvolume” law asymptotic formula in the random-matrix case. Here is another indication provided by the case L=N−1, i.e., (49), with $\delta_{N}=N^{-1}$. In this case, it is possible to find an exact asymptotic formula valid with a probability exceeding 1−ε for any ε>0, i.e., for the overwhelming majority of realizations (see Appendix D):

(59) $$S_{LN}{|}_{L=N-1}=h\left(\kappa \right)+o\left(1\right),\;N\to \infty ,$$

i.e., we have a formal analog of the one-dimensional area law.

In particular (cf. (52) and (58)),

$$S_{LN}{|}_{L=N-1,\kappa =1/2}=1+o\left(1\right),\;N\to \infty .$$

In addition, we have in this case ${\left(4\kappa \left(1-\kappa \right)+O\left(1/N\right)\right)|}_{\kappa =1/2}=1+O\left(1/N\right),$ i.e., the lower bound (50) of the entanglement entropy coincides with its value for the overwhelming majority of realizations.

### 2.3. Entanglement Entropy of Hawking Radiation

The problem is as follows. Viewing a black hole and its radiation as a bipartite quantum system (1) and (2), denote

(60) $$\dim H_{B}=L,\;\dim H_{E}=K,\;\dim H_{S}=\mathrm{KL}=N$$

and index the bases in the state spaces $H_{B}$ and $H_{E}$ of its parties by l=1,…,L and k=1,…,K.

Note that in the discussed above case of free fermions, where the description reduces to the one-body picture, the indexing sets of the block and its environment are Λ and Ω∖Λ, but in that case, |Ω|=|Λ|+|Ω∖Λ|, while in (60), we have N=KL. This is because the second quantization is a kind of the “exponentiation” of the one-body picture.

Assuming the complete ignorance of the structure of the whole system S (an evaporating black hole and its radiation), one can choose as its ground state

(61) $$|\Psi_{S}\rangle ={\left\{\Psi_{kl}\right\}}_{k,l=1}^{K,L},$$

the random vector uniformly distributed over the unit sphere in $H_{S}=H_{B}⊗H_{E}=C^{N},$N=KL (see (2) and (60)). Thus, the density matrix $\rho_{N}$ of S and the reduced density matrix $\rho_{\mathrm{LN}}$ of B (the radiation) are

(62) $${\left(\rho_{N}\right)}_{k_{1}l_{1},k_{2}l_{2}}=\Psi_{k_{1}l_{1}}\bar{\Psi_{k_{2}l_{2}}},\;\;{\left(\rho_{\mathrm{LN}}\right)}_{l_{1}l_{2}}=\sum_{k=1}^{K}\Psi_{kl_{1}}\bar{\Psi_{kl_{2}}},$$

(cf. (3) and (4)).

It is of interest to find the typical behavior of the corresponding (random) entanglement entropy (see (5)). It was suggested in [34], as a first step in this program, that

(63) $$E\left\{S_{\mathrm{LN}}\right\}=\sum_{t=K+1}^{\mathrm{KL}}\frac{1}{t}-\frac{L-1}{2K},\;L\leq K=N/L.$$

Formula (63) was then proved by using an explicit and rather involved form of the joint eigenvalue distribution of random matrix $\rho_{\mathrm{LN}}$ (62): see, e.g., [6,29] for reviews.

It follows from (63) that the two-term asymptotic formula for large K values and any L, i.e., for

(64) 1≲L≪K≲N

(cf. (6)), is

(65) $$E\left\{S_{\mathrm{LN}}\right\}=\log L-\frac{L}{2K}+O\left(1/K\right),\;K=N/L\to \infty .$$

Note that here we follow [34] and use the (cf. (6)) standard natural log:=ln to the base e= 2.7182 instead of $\log_{2}$, as in the definition (5) of the von Neumann entropy.

It follows from (63) that in the asymptotic regime of successive limits (first K→∞, then L→∞ (cf. (8) and (64)), we have

(66) $$\lim_{K\to \infty }E\left\{S_{\mathrm{LN}}\right\}:=E\left\{S_{L}\right\}=\log L+o\left(1\right),\;L\to \infty .$$

Moreover, the same holds in the asymptotic regime of simultaneous limits K→∞, L→∞, provided that L/K=o(1), i.e., 0≤α<1 (cf. (22)).

This case can be viewed as that describing the very initial stage of black hole radiation. On the other hand, in the asymptotic regime (cf. (22) and (51))

(67) K→∞,L→∞,L/K→ℓ>0,

another possible implementation of the analog of the heuristic inequalities (6) (cf. (22) with α=1), we have (cf. (7))

(68) $$E\left\{S_{\mathrm{LN}}\right\}=\log L-\left\{\begin{array}{cc} \ell /2, & 0\leq \ell \leq 1,\\ 1/2\ell +\log\ell , & \ell \geq 1. \end{array}\right.$$

This case corresponds to a later stage of black hole radiation.

It can also be shown that the fluctuations of $S_{\mathrm{LN}}$ vanish for large K and L values: see, e.g., [6].

Based on the Formula (68), an interesting scenario of black hole evaporation was proposed in [9,34]: see also [2] for a recent review.

Here, we only mention that the function given by the r.h.s. of (68) (see Figure 4) is a monotone-increasing, convex, and piece-wise analytic. Its *p*th derivative has a jump from 0 to ${\left(-1\right)}^{p}\left(p-1\right)!\left(p/2-1\right)$; for all p≥3, “a phase transition” of the third order takes place.

Recall that the maximum of the von Neumann entropy (5) over the set of T×T positive definite matrices of trace 1 is equal to $\log_{2}T$. We conclude, following [34], that while the (random) states (61) (see also (73)) of the whole system are pure, the subsystem states are typically quite close to the maximally mixed states with the “deficit” given by the second term of the r.h.s. of (68).

The link of the above results with those of the previous subsection is as follows. It was mentioned there that in the case of free fermions, the dimension $\dim H_{S}$ of the state space of the system S and the volume $\left|\Omega \right|={\left|N\right|}^{d}$ of the domain occupied by S are related as $\left|\Omega \right|={\left|N\right|}^{d}=\log_{2}\dim H_{S}$ and the same for its party B, occupying a subdomain $\Lambda :L^{d}=\left|\Lambda \right|=\log_{2}\dim H_{B}$. In fact, this logarithmic dependence is general for the many-body quantum systems. Thus, viewing the black hole and its radiation as the parties of a many-body bipartite system (see (1)) and taking into account (60), we can interpret logL in the asymptotic Formulas (66)–(68) as the “volume” of the spatial domain occupied by the black hole radiation; hence, these asymptotic formulas are the analogs of the volume law (11): see (45) and (52) in particular.

We will show now that the standard facts of random matrix theory, that date back to the 1960s, provide a streamlined proof of the validity of (66)–(68) for a rather wide class of random vectors, including those of (61), and not only for the expectation of the entanglement entropy but also for its all-typical realization, i.e., with probability 1. One can say that these results manifest the typicality and the universality of Page’s formula, given by the r.h.s. of (68) and (75). For other versions of these important properties, see [6,29,46].

Let

(69) $${\left\{X_{lk}\right\}}_{l,k=1}^{\infty },\;E\left\{X_{lk}\right\}=E\left\{X_{lk}^{2}\right\}=0,\;E\{|X_{lk}{|}^{2}\}=\xi^{2}>0$$

be an infinite collection of independent identically distributed (i.i.d.) complex random variables with zero mean and unit variance,

(70) $$X_{LN}={\left\{X_{lk}\right\}}_{l,k=1}^{L,K}$$

be the K×L matrix, and

(71) $$Z_{LN}=\mathrm{Tr}X_{LN}X_{LN}^{*}=\sum_{l=1}^{L}\sum_{k=1}^{K}{\left|X_{lk}\right|}^{2}.$$

View $X_{LN}$ as a random vector in

(72) $$C^{N}=C^{K}⊗C^{L},\;N=\mathrm{KL},$$

and (71) as the square of its Euclidian norm, and introduce the corresponding random vector of unit norm (cf. (61))

(73) $$\Psi_{N}=X_{LN}/Z_{LN}^{1/2}.$$

Note that if ${\left\{X_{kl}\right\}}_{k,l=1}^{\infty }$ are the complex Gaussian random variables with zero mean and unit variance, then $\Psi_{N}$ of (73) is uniformly distributed over the unit sphere of $C^{N}$ (see (72)), hence coinciding with (61) and the setting of [34].

**Result 4.**  *Consider a bipartite quantum system having the random vector (73) as its ground state. Let $S_{\mathrm{LN}}$ be the entanglement entropy defined by (5), and (62) with the $\Psi_{N}$ of (73). Then, we have the analogs of (66)–(68) valid for all typical realizations of (73) (with probability 1, with respect to (69)):*

(74) $$\lim_{K\to \infty }S_{\mathrm{LN}}:=S_{L}=\log L+o\left(1\right),\;L\to \infty ,$$

*and*

(75) $$S_{\mathrm{LN}}=\log L-\left\{\begin{array}{cc} \ell /2, & 0\leq \ell \leq 1,\\ 1/2\ell +\log\ell , & \ell \geq 1, \end{array}\right.+o\left(1\right),\;\;K,L\to \infty ,\;L/K\to \ell >0.$$

One can say that these results manifest the typicality (the validity with probability 1) and the universality (the independence of the probability law of $\left\{X_{jk}\right\}$) in (69)) of Page’s formula, given by the r.h.s. of (68) and (75). For other versions of these important properties, see [6,29,46].

The proof of this result is given in Appendix E.

## 3. Conclusions

Our main motivation was to study the possible asymptotic forms of the entanglement entropy of quantum bipartite systems in a regime where the size of one of the parties (a block) grows simultaneously with the size of the system. We believe that this regime is of interest both in itself and because it seems more adequate for interpreting numerical results. This regime can be considered for various cases of interaction radii and hopping in the Hamiltonian of the system.

Using a random matrix as a one-body Hamiltonian can serve as a model for long-range hopping that has a radius of the same order of magnitude as the size of the system. We show that in this case, the asymptotic behavior of the entanglement entropy follows the volume law, but not the area law or the enhanced area law, that arises in the case of finite-range hopping and the widely used asymptotic regime in which the block size is considered large only after a macroscopic limit passage for the entire system.

For the proof, we use both new, seemingly quite general two-sided bounds for the entanglement entropy and existing rigorous results from random matrix theory. The latter also proved to be useful for analyzing the generalization of the Hawking radiation model in the theory of black holes. This analysis, which turns out to be fairly simple and transparent, is also presented in this paper. It implies the validity of the Page formula, obtained initially for a particular case, in a fairly wide class of typical random states of the system.

## Figures and Tables

**Table 1:** Closed-form expressions for the coefficient $s_{\kappa \lambda }$ of (52)–(55).

№	Condition	$s_{\kappa \lambda }$
I	λ<κ	$-\frac{1}{\ln(2)}\left\{\bar{\kappa }\ln\left(\bar{\kappa }\right)+\kappa \ln\left(\kappa \right)+\frac{\bar{\lambda }}{\lambda }\ln\left(\bar{\lambda }\right)+1\right\}$
	$\lambda <\bar{\kappa }$	
II	λ<κ	$-\frac{\bar{\kappa }}{\ln(2)}\left\{\frac{\bar{\lambda }}{\lambda }\ln\left(\bar{\lambda }\right)+\ln\left(\lambda \right)+\frac{\kappa }{\bar{\kappa }\lambda }\ln\left(\kappa \right)+1\right\}$
	$\lambda >\bar{\kappa }$	
III	λ>κ	$-\frac{\kappa }{\lambda \ln(2)}\left\{\lambda \ln\left(\lambda \right)+\bar{\lambda }\ln\left(\bar{\lambda }\right)+\frac{\bar{\kappa }}{\kappa }\ln\left(\bar{\kappa }\right)+1\right\}$
	$\lambda <\bar{\kappa }$	
IV	λ>κ	$-\frac{1}{\lambda \ln(2)}\left\{\bar{\lambda }\left[\bar{\kappa }\ln\left(\bar{\kappa }\right)+\kappa \ln\left(\kappa \right)\right]+\lambda \ln\left(\lambda \right)+\bar{\lambda }\right\}$
	$\lambda >\bar{\kappa }$	

**Table 2:** Maximal distances between the coefficients $C_{\pm }$ of (48) and $s_{\kappa \lambda }$ of (53). Left: $\Delta \left(C_{-},s\right)=\max_{\lambda }\left[s-C_{-}\right]$, $\Delta \left(s,C_{+}\right)=\max_{\lambda }\left[C_{+}-s\right]$, and $\Delta \left(C_{-},C_{+}\right)=\max_{\lambda }\left[C_{+}-C_{-}\right]$ at different values of κ. Right: the same distances but with $\max_{\kappa }$ for different values of λ.

κ	$\Delta (C_{-},s)$	$\Delta (s,C_{+})$	$\Delta (C_{-},C_{+})$	λ	$\Delta (C_{-},s)$	$\Delta (s,C_{+})$	$\Delta (C_{-},C_{+})$
0.1	0.10	0.09	0.1	0.1	0.07	0.04	0.1
0.2	0.08	0.096	0.1	0.2	0.06	0.07	0.1
0.3	0.05	0.08	0.1	0.3	0.06	0.09	0.1
0.4	0.05	0.08	0.1	0.4	0.06	0.09	0.1
0.5	0.06	0.09	0.1	0.5	0.06	0.09	0.1
0.6	0.06	0.08	0.1	0.6	0.04	0.09	0.1
0.7	0.05	0.08	0.1	0.7	0.02	0.08	0.1
0.8	0.08	0.09	0.1	0.8	0.014	0.09	0.1
0.9	0.1	0.09	0.1	0.9	0.01	0.07	0.07

## Data Availability

The raw data supporting the conclusions of this article will be made available by the authors on request.

## Appendix A. Proof of Result 1

This proof is based on the following properties of *h* and $h_{0}$ of (19) and (26), which are easy to check (see, e.g., [20]):

(i) *h* is concave ($h^{\prime \prime }\left(x\right)\leq 0,\;x\in \left(0,1\right)$), and

(A1) h(x)≥4x(1−x),x∈[0,1];

(ii) $h_{0}$ of (26) is also concave ($h_{0}^{\prime \prime }\left(y\right)\leq 0,\;y\in \left(0,1/4\right)$)), and

(A2) $$h_{0}\left(y\right)=-y\log_{2}y+O\left(y\right),\;y↘0.$$

To get the lower bound in (24), we denote by ${\left\{p_{\alpha }\right\}}_{\alpha =1}^{\left|\Lambda \right|}$ the eigenvalues of $P_{LN}$ of (17) and use (A1) and (25):

$$\begin{array}{cc} \; & \;S_{LN}=\mathrm{Tr}h\left(P_{LN}\right)=\sum_{\alpha =1}^{\left|\Lambda \right|}h\left(p_{\alpha }\right) \end{array}$$

(A3) $$\begin{array}{c} \;\;\;\;\;\;\;\;\;\;\;\;\geq \sum_{\alpha =1}^{\left|\Lambda \right|}4p_{\alpha }\left(1-p_{\alpha }\right)=4\mathrm{Tr}P_{LN}\left(1_{\Lambda }-P_{LN}\right)=:L_{LN}. \end{array}$$

To get the upper bound in (24), we use (25) (or (A3)), (26), and the concavity of $h_{0}$, implying by the Jensen inequality

$$\begin{array}{cc} \; & \;S_{LN}=\sum_{\alpha =1}^{\left|\Lambda \right|}h\left(p_{\alpha }\right)=\sum_{\alpha =1}^{\left|\Lambda \right|}h_{0}\left(p_{\alpha }\left(1-p_{\alpha }\right)\right)\\ \; & =|\Lambda |\;({\left|\Lambda \right|}^{-1}\sum_{\alpha =1}^{\left|\Lambda \right|}h_{0}(p_{\alpha }\left(1-p_{\alpha }\right))\leq |\Lambda |\;h_{0}({\left(4|\Lambda |\right)}^{-1}\;L_{\Lambda \Omega })=:U_{LN}. \end{array}$$

To obtain (27) and (28), we just apply the expectation to (24) and use once more the Jensen inequality in the r.h.s.:

$$E\left\{U_{LN}\right\}=|\Lambda |E\{h_{0}\left({\left(4|\Lambda \right)}^{-1}|L_{LN}\right\}\leq \left|\Lambda \right|h_{0}\left({\left(4|\Lambda \right)}^{-1}|E\left\{L_{LN}\right\}\right).$$

## Appendix B. Proof of Result 2

We will use the bounds (24)–(26). It follows from (39) that the expectation of the lower bound $L_{LN}$ is expressed via the mixed fourth moments of the entries ${\left\{U_{kl}\right\}}_{k,l=1}^{N}$ of the Haar distributed unitary matrix $U_{N}$. These moments are known as follows (see, e.g., [32], Problem 8.5.2):

(A4) $$\begin{array}{cc} \; & \;E\{U_{a_{1}b_{1}}{\bar{U}}_{\alpha_{1}\beta_{1}}U_{a_{2}b_{2}}{\bar{U}}_{\alpha_{2}\beta_{2}}\}\\ \; & ={\left(n^{2}-1\right)}^{-1}\left(\delta_{a_{1}\alpha_{1}}\delta_{b_{1}\beta_{1}}\delta_{a_{2}\alpha_{2}}\delta_{b_{2}\beta_{2}}+\delta_{a_{1}\alpha_{2}}\delta_{b_{1}\beta_{2}}\delta_{a_{2}\alpha_{1}}\delta_{b_{2}\beta_{1}}\right)\\ \; & -{\left(n\left(n^{2}-1\right)\right)}^{-1}\left(\delta_{a_{1}\alpha_{2}}\delta_{b_{1}\beta_{1}}\delta_{a_{2}\alpha_{1}}\delta_{b_{2}\beta_{2}}+\delta_{a_{1}\alpha_{1}}\delta_{b_{1}\beta_{2}}\delta_{a_{2}\alpha_{2}}\delta_{b_{2}\beta_{1}}\right), \end{array}$$

and we obtain for 1≤l<m≤N

$$E\{|P_{lm}{|}^{2}\}=\sum_{k_{1},k_{2}=1}^{K}E\left\{U_{lk_{1}}U_{mk_{2}}\bar{U_{mk_{1}}}\;\bar{U_{lk_{2}}}\right\}=\frac{1}{\left(N^{2}-1\right)}\sum_{k_{1},k_{2}=1}^{K}\left(\delta_{k_{1}k_{2}}-N^{-1}\right)\frac{K(N-K)}{N(N^{2}-1)}.$$

This and (25) imply that

(A5) $$E\left\{L_{LN}\right\}=4\sum_{l=1}^{L}\sum_{m=L+1}^{N}E\{|P_{lm}{|}^{2}\}=4\frac{K(N-K)L(N-L)}{N(N^{2}-1)},$$

and then (24) and (40) yield (45).

## Appendix C. Proof of Result 3

We will use Formulas (29) and (30), which reduce the problem of the asymptotic analysis of entanglement entropy to that of the Counting Measure $N_{P_{LN}}$ (29) of the random matrix $P_{LN}$ (42) in the regime (44): more precisely, the limit

(A6) $$\nu_{P}=\lim\;N_{P_{LN}}/L$$

in the sense of (44) (see also Appendix E for a similar approach).

The explicit form of $\nu_{P}$ has actually been known since 1980 and is called the Wachter distribution. It was obtained in [47] in the context of statistics, and according to this work, convergence in (A6) is in probability. In the subsequent works [32,48,49,50], the distribution was obtained through other methods and in other settings, in particular, the convergence with probability 1 was also proven.

We have, according to these works,

(A7) $$\begin{array}{cc} \; & \;\nu_{P}=m_{0}\delta_{0}+m_{1}\delta_{1}+\nu_{ac},\\ m_{0} & =\max\left(\lambda -\kappa ,0\right)/\lambda ,\;m_{1}=\max\left(\lambda +\kappa -1,0\right)/\lambda , \end{array}$$

and the density $\nu_{ac}^{\prime }$ of $\nu_{ac}$ in (A7) is given by (54). Now, plugging $\nu_{P}$ into the divided-by-*L* version of (30) for our case, and assuming (44), we obtain (52)–(54), taking into account that the atoms in (A7) do not contribute to the integral in the r.h.s. of the limiting form of (30) because of equalities h(0)=h(1)=0.

Note that the atom at 0 in (A7) can be obtained just by calculating the rank of (42) (cf. (A24)) and the atom at 1 has the same origin, because we have the following in view of the unitarity of $U_{N}$:

(A8) $${\left(P_{LN}\right)}_{l_{1}l_{2}}=\sum_{k=1}^{K}U_{l_{1}k}\bar{U_{kl_{2}}}=\delta_{l_{1}l_{2}}-\sum_{k=K+1}^{N}U_{l_{1}k}\bar{U_{kl_{2}}}.$$

## Appendix D. Proof of (59)

It follows from (18), (19) and (26) that (see also (32))

(A9) $$S_{LN}=\mathrm{Tr}h_{0}\left(P_{LN}\left(1_{\Lambda }-P_{LN}\right)\right).$$

It is easy to see that if L=N−1, then

$${\left(P_{LN}\left(1_{\Lambda }-P_{LN}\right)\right)}_{l_{1}l_{2}}={\left(P_{\Omega }\right)}_{l_{1}N}\bar{{\left(P_{\Omega }\right)}_{l_{2}N}},\;l_{1},l_{2}=1,\ldots ,\left(N-1\right),$$

i.e., it is a hermitian operator of rank one. Hence, its eigenvalues are 0 of multiplicity (L−1) and

$$q_{N}=\sum_{l=1}^{N-1}{\left|{\left(P_{\Omega }\right)}_{lN}\right|}^{2}$$

of multiplicity 1. This, (39), and the unitarity of $U_{N}$ imply that

(A10) $$q_{N}=u_{N}\left(1-u_{N}\right),\;u_{N}=\sum_{l=1}^{N-1}{\left|U_{lN}\right|}^{2},$$

and then, through (A9),

$$S_{LN}{|}_{L=N-1}=h_{0}\left(u_{N}\left(1-u_{N}\right)\right).$$

It follows then from the explicit form of the forth mixed moments of the entries ${\left\{U_{lk}\right\}}_{l,k=1}^{N}$ of the Haar distributed matrix $U_{N}$ (see (A4)) that

$$\begin{array}{cc} \; & \;{\bar{u}}_{N}:=E\left\{u_{N}\right\}=K/N=\kappa \left(1+O\left(1/N\right)\right),\\ \; & \mathrm{Var}\left\{u_{N}\right\}:=E\{|u_{N}-{\bar{u}}_{N}{|}^{2}{\}=E\{|u-K/N|}^{2}\}\leq \kappa \left(1-\kappa \right)N^{-1}\left(1+O\left(1/N\right)\right),\;N\to \infty . \end{array}$$

Hence, according to the Tchebychev inequality, we have for any ε>0

(A11) $$P\{|u_{N}-K/N\left|>\varepsilon \right\}\leq \mathrm{Var}\left\{u_{N}\right\}/\varepsilon^{2}\leq \kappa \left(1-\kappa \right)/\varepsilon^{2}N.$$

We conclude via the continuity of $h_{0}$ that for the overwhelming majority of realizations (with probability 1−ε for any ε>0), we have

(A12) $$S_{LN}{|}_{L=N-1}=h_{0}\left(\kappa \left(1-\kappa \right)\right)+o\left(1\right),\;\varepsilon \to 0.\;$$

For instance, we can choose $\varepsilon =N^{-1/3}$ to have $O(1/N^{1/3})$ in the r.h.s. of (A11) and (A12).

## Appendix E. Proof of Result 4

Following (61)–(62) and (69)–(73), we obtain the following for the corresponding reduced density matrix:

(A13) $$\rho_{LN}=X_{LN}X_{LN}^{*}/Z_{LN}.$$

Dividing the numerator and denominator by $\xi^{2}K$, we obtain

(A14) $$\rho_{LN}=W_{LN}/Y_{LN},$$

where

(A15) $$\begin{array}{cc} \; & W_{LN}=X_{LN}^{*}X_{LN}/K,\;Y_{LN}=Z_{LN}/K,\;Z=\sum_{l,k=1}^{L,K}{\left|X_{lk}\right|}^{2}, \end{array}$$

and ${\left\{X_{lk}\right\}}_{l,k=1}^{\infty }$ are independent identically distributed random variables, such that (cf. (69))

(A16) $$E\left\{X_{lk}\right\}=E\left\{X_{lk}^{2}\right\}=0,\;E\{|X_{lk}{|}^{2}\}=1.$$

This allows us to write the von Neumann entropy of (A14) as

(A17) $$\begin{array}{cc} \; & S_{LN}=\log Y_{LN}-Y_{LN}^{-1}\mathrm{Tr}W_{LN}\log W_{LN}\\ \; & \;=\log Y_{LN}-Y_{LN}^{-1}\int w\log w\;\nu_{W_{LN}}\left(dw\right), \end{array}$$

where

(A18) $$\nu_{W_{LN}}=N_{W_{LN}}/L$$

is the Normalized Counting Measure of eigenvalues of $W_{LN}$ (cf. (29)).

It follows from the Strong Law of Large Numbers for the collection (69) that we have with probability 1 for any L and K→∞

(A19) $$Z_{LN}=\sum_{k,l=1}^{K,L}{\left|X_{kl}\right|}^{2}=\mathrm{KL}\left(1+o\left(1\right)\right),\;N:=\mathrm{KL}\to \infty .$$

Thus, the first term on the right of (A17) is, in view of (A15),

(A20) $$\log Y_{LN}=\log L+o\left(1\right),\;K\to \infty ,$$

i.e., it coincides with that in (65). Note that this is valid in both of the asymptotic regimes, the subsequent limits K→∞ and then L→∞ and the simultaneous limits (67), and for all typical realizations (with probability 1).

Consider now the second term on the right of (A17) and assume first that L is fixed while K→∞. Since (see (A15))

(A21) $${\left(W_{LN}\right)}_{l_{1}l_{2}}=K^{-1}\sum_{k=1}^{K}X_{l_{1}k}X_{l_{2}k}^{*},\;l_{1},l_{2}=1,\ldots ,K,$$

it follows, again from the Strong Law of Large Numbers and (69), that $W_{LN}$ converges with probability 1 as K→∞ to the L×L unit matrix $1_{L}$; thus, the second term on the right of (A17) vanishes as K→∞. We conclude that in the regime of successive limits (see (67)), we have with probability 1

(A22) $$S_{L}:=\lim_{K\to \infty }S_{LN}=\log L,$$

i.e., the version of (66) but now for all typical realizations and for the not-necessarily Gaussian $X_{LN}$ of (70) with i.i.d. components (69). In the context of the black hole model of [9,34], this result corresponds to the maximum mixed state of the Hawking radiation despite the pure initial state (or (61) of the whole system (black hole and radiation).

Passing to the regime of simultaneous limits (67), we note first that the matrix $W_{LN}$ has been known in statistics since the late 1920s as the sample covariance matrix of the sample of K random L-dimensional vectors $X_{k}={\left\{X_{lk}\right\}}_{l=1}^{L},\;k=1,\ldots ,K$ (data vectors), and according to [51] (see also [32] for a review), the Normalized Counting Measure $\nu_{W_{LN}}$ (A18) of eigenvalues of $W_{LN}$ converges with probability 1 to the non-random limit (cf. (54) and (A7))

(A23) $$\begin{array}{cc} \; & \lim_{K\to \infty ,L\to \infty ,L/K\to \ell }\nu_{W_{LN}}=:\nu_{W}=\max\left\{1-\ell ,0\right\}\delta_{0}+\nu_{ac},\\ \; & \nu_{ac}^{\prime }\left(w\right)=\frac{\sqrt{\left(w_{+}-w\right)\left(w-w_{-}\right)}}{2\pi \ell w}\chi_{\left[w_{-},w_{+}\right]},\;w_{\pm }={\left(1\pm \sqrt{\ell }\right)}^{2}, \end{array}$$

where χ is the same as that defined in (14).

Note that the atom at 0 in (A23) is just (cf. (A8))

(A24) $$\lim_{K\to \infty ,L\to \infty ,L/K\to \ell }\left(L-\mathrm{rank}\;W_{\mathrm{LN}}\right)/L.$$

It follows from (A18)–(A20) and (A23) that the simultaneous limit (67) of the second term in (A17) is

(A25) $$I=\frac{1}{2\pi \ell }\int_{w_{-}}^{w_{+}}\sqrt{\left(w-w_{+}\right)\left(w-w_{-}\right)}\;\log wdw.$$

We will use the identity

$$\log w=\int_{0}^{\infty }\left(\frac{1}{t+1}-\frac{1}{t+w}\right)dt,$$

implying that

(A26) $$I=\lim_{A\to \infty }\left(I_{1}-I_{2}\right),$$

where

(A27) $$\begin{array}{ccc} I_{1} & = & \frac{1}{2\pi \ell }\int_{0}^{A}\frac{dt}{t+1}\int_{w_{-}}^{w_{+}}\sqrt{\left(w-w_{+}\right)\left(w-w_{-}\right)}dw,\;\\ I_{2} & = & \frac{1}{2\pi \ell }\int_{0}^{A}dt\int_{w_{-}}^{w_{+}}\frac{\sqrt{\left(w-w_{+}\right)\left(w-w_{-}\right)}}{t+w}dw. \end{array}$$

By changing variables in the integral over *w* in $I_{1}$ to

(A28) $$w=m+lx,\;m=\frac{w_{+}+w_{-}}{2}=\left(1+\ell \right),\;l=\frac{w_{+}-w_{-}}{2}=2\sqrt{\ell },$$

we obtain

(A29) $$I_{1}=\frac{2}{\pi }\int_{0}^{A}\frac{dt}{t+1}\int_{-1}^{1}\sqrt{1-x^{2}}dx=\int_{0}^{A}\frac{dt}{t+1}=\log A+O\left(1\right),\;A\to \infty .$$

The same change of variables (A28) yields

$$I_{2}=\frac{l}{2\pi \ell }\int_{0}^{A}dt\int_{-1}^{1}\frac{\sqrt{1-x^{2}}}{x+\tau }dx,\;\tau =\frac{t+m}{l}>1.$$

The integral over *x* is $\pi (\tau -\left(\tau^{2}-1\right))$; hence, with (A28),

(A30) $$\begin{array}{ccc} I_{2} & = & 2\int_{m/l}^{(A+m)/l}\left(\tau -{\left(\tau^{2}-1\right)}^{1/2}\right)d\tau \\ & = & {\left.\left(\frac{1}{2}\log\left(\tau +{\left(\tau^{2}-1\right)}^{1/2}\right)-\frac{1}{4}{\left(\tau -{\left(\tau^{2}-1\right)}^{1/2}\right)}^{2}\right)\right|}_{\tau =m/l}^{(A+m)/l}. \end{array}$$

Now, taking into account (A28),

(A31) $$\begin{array}{ccc} {\left.\left(\tau +{\left(\tau^{2}-1\right)}^{1/2}\right)\right|}_{\tau =m/l} & = & 2l^{-1}\max\left(1,\ell \right),\\ {\left.\left(\tau -{\left(\tau^{2}-1\right)}^{1/2}\right)\right|}_{\tau =m/l} & = & 2l^{-1}\min\left(1,\ell \right),\\ {\left.\left(\tau +{\left(\tau^{2}-1\right)}^{1/2}\right)\right|}_{\tau =(A+m)/l} & = & 2Al^{-1}+O\left(1/A\right),\;A\to \infty , \end{array}$$

and (A26)–(A31), we obtain the following for *I* of (A25):

(A32) $$I=2^{-1}\min\left(\ell ,\ell^{-1}\right)+\log\;\max\left(1,\ell \right).$$

This coincides with the second term of the r.h.s. of (68), and hence proves Result 4 with probability 1.
